# Effect of photobiomodulation therapy on regulating reparative processes of chronic wounds at the inflammation stage

**DOI:** 10.7705/biomedica.7820

**Published:** 2026-08-20

**Authors:** Rostislav Shevchenko, Sergey Pavlov, Nataliia Babenko, Marina Kumetchko, Olga Litvinova, Inna Torianyk

**Affiliations:** 1 Department of Surgery N°4, Kharkiv National Medical University, Kharkiv, Ukraine Department of Surgery N°4 Kharkiv National Medical University Kharkiv Ukraine; 2 Laboratory of Antimicrobial Agents, I. Mechnikov Institute of Microbiology and Immunology, National Academy of Medical Sciences of Ukraine, Kharkiv, Ukraine Institute of Microbiology and Immunology National Academy of Medical Sciences of Ukraine Kharkiv Ukraine

**Keywords:** Low-level light therapy, wound healing, inflammation, platelet aggregation, interleukins, terapia por luz de baja intensidad, cicatrización de heridas, inflamación, agregación plaquetaria, interleucinas

## Abstract

**Introduction.:**

Chronic wounds remain a major burden for patients and healthcare systems worldwide. Photobiomodulation therapy has demonstrated effectiveness as a physical method to support wound healing.

**Objective.:**

To assess whether photobiomodulation, applied with specific parameters, can modulate the inflammatory microenvironment of chronic wounds by examining its effects on pro and antiinflammatory cytokines, key growth factors, reactive oxygen species levels, and platelet aggregation, integrating these results with histopathological findings.

**Materials and methods.:**

Eighteen rats were randomized into "intact", "control", and "experimental" groups. Chronic wounds were induced in control and experimental groups, with the latter receiving photobiomodulation therapy. Animals were euthanized 3 days postsurgery. Serum levels of reactive oxygen species (ROS), IL-1ß, IL4, IL6, IL10, TNFα, bFGF, and TGF-ß1 were measured via ELISA. Platelet aggregation assays and histological examinations were conducted.

**Results.:**

On day 3, photobiomodulation increased ROS levels in the experimental group by 1.24 fold compared with controls and by 1.23 fold compared with intact rats. Photobiomodulation reduced IL4 levels by 1.58 fold and decreased IL6 levels twofold relative to controls. While IL6 increased in control animals compared with intact rats, photobiomodulation reduced IL6 below intact levels. No significant differences were observed for IL1-ß, IL10, TNFα, bFGF, or TGF-ß1. Photobiomodulation was associated with reduced platelet aggregation during the inflammatory phase. Histological analysis revealed fewer macrophages and polymorphonuclear leukocytes and a higher number of fibroblasts in photobiomodulationtreated wounds.

**Conclusion.:**

Photobiomodulation therapy positively influences chronic wound healing by beneficially modulating inflammatory processes and promoting a more favorable tissue repair environment.

## Resumen

**Introducción.:**

Las heridas crónicas siguen siendo una carga importante para los pacientes y los sistemas de salud en todo el mundo. La terapia con fotobiomodulación ha demostrado ser eficaz como método físico para la cicatrización.

**Objetivo.:**

Evaluar si la fotobiomodulación, aplicada con parámetros específicos, puede modular favorablemente el microambiente inflamatorio de las heridas crónicas mediante el análisis de citocinas pro y antiinflamatorias, factores de crecimiento clave, niveles de especies reactivas de oxígeno y actividad de agregación plaquetaria, correlacionando estos resultados con los hallazgos histopatológicos.

**Materiales y métodos.:**

Se asignaron aleatoriamente 18 ratas a los grupos «intacto», «control» y «experimental». Se indujeron heridas crónicas en los grupos control y experimental; este último recibió fotobiomodulación. Las ratas se sacrificaron tres días después. Se midieron en suero las especies reactivas de oxígeno, IL-1 ß, IL4, IL6, IL10, TNFα, bFGF y TGF-ß1 mediante ELISA. Se realizaron estudios de agregación plaquetaria y análisis histológico.

**Resultados.:**

Al día 3, la fotobiomodulación aumentó los niveles de especies reactivas de oxígeno en el grupo experimental 1,24 veces respecto al grupo control y 1,23 veces respecto al grupo intacto. El tratamiento redujo la IL4 en un 1,58 y disminuyó la IL6 a la mitad respecto al grupo control, mientras que la IL6 aumentó en el grupo control frente al grupo intacto. No se observaron diferencias significativas en IL-1 ß, IL10, TNFα, bFGF ni TGF-ß1. La fotobiomodulación se asoció con menor agregación plaquetaria durante la fase inflamatoria. Histológicamente, en las heridas tratadas se encontraron menos macrófagos y leucocitos polimorfonucleares y un mayor número de fibroblastos.

**Conclusión.:**

La fotobiomodulación influye positivamente en la cicatrización de las heridas crónicas al modular de forma beneficiosa los procesos inflamatorios.

Chronic wounds continue to be a heavy burden for patients and medicine in all countries. According to the World Health Organization (WHO), chronic wounds affect 2% of the world's population ^1^. Chronic wound formation can be influenced by many conditions, such as aging, comorbidities, endocrine factors, obesity, and stress ^2^^,^^3^.

Wound healing results from sequential, overlapping, highly coordinated stages: hemostasis, inflammation, proliferation, and remodeling. The inflammation stage is a key stage of wound healing as it determines the healing progression ^4^. However, chronic wounds often exhibit prolonged inflammation and delayed or impaired transition to the proliferation phase ^5^.

Understanding the processes of wound formation and healing at the cellular and molecular level will enable us to find effective methods for stimulating the repair process. Modern wound treatment methods, such as nanotechnology ^6^, xenogeneic drugs ^7^, physical methods of influence, etc., are aimed, among other things, at resolving inflammation.

Photobiomodulation therapy has shown its effectiveness among physical methods in wound treatment. This therapy is also used to treat many diseases, such as rheumatoid arthritis ^8^, cognitive impairment, and to reduce pain ^9^.

The molecular mechanisms of the therapeutic effect of photobiomodulation are associated with intracellular effects on mitochondrial cytochrome C oxidase. They include light-sensitive transporters and cell membrane receptors or extracellular activation of transforming growth factor-ß1 (TGF-ß1), which may contribute to subsequent reactions involving signaling molecules ^10^.

The positive photobiomodulation effects in the red and near-infrared spectral band ranges are related to the generation of ROS, which increases ATP production and stimulates enzymes and transcription factors ^11^. Exposure to red light emitted by lasers or light-emitting diodes (LED) also affects the functional activity of platelets ^12^.

The use of photobiomodulation therapy in the early stages of chronic wound healing helps to reduce inflammation, decrease the number of newly formed vessels, and improve the organization of collagen fibers during the remodeling stage ^13^. This therapy can optimize reparative processes by correcting the regulation by intercellular mediators ^14^.

Despite its clinical efficacy, the precise molecular mechanisms by which photobiomodulation regulate the inflammatory phase in chronic wounds are not fully understood. This is particularly relevant when assessing the complex effects of photobiomodulation on platelet activity, oxidative stress, and cytokine profiles. In addition, further evidence is needed to optimize the parameters of this therapy.

The purpose of this work was to evaluate whether photobiomodulation, applied with specific parameters (660 nm, 2 J/cm^2^), can favorably modulate the inflammatory microenvironment of chronic wounds by analyzing its impact on the balance of pro- and anti-inflammatory cytokines (IL-1 ß, IL-4, IL-6, IL-10, TNF-α), the expression of key growth factors (bFGF, TGF-ß1), the levels of ROS, and platelet aggregation activity, correlating them with histopathological findings.

## Materials and methods

The study was conducted on 18 Wistar albino Glaxo rats, weighing about 230 g, aged 9-10 months. The animals were bred and kept at the Kharkiv National Medical University vivarium in compliance with all sanitary and hygienic standards. The animals were randomized into three groups: intact, control, and experimental, each consisting of 6 rats.

Chronic wounds were modeled under anesthesia (zoletil mono narcosis at the rate of 10 mg/kg body weight) in animals of control and experimental groups, with reproduction of conditions of microcirculation disturbance and hypoxia ^15^. The wounds were circular, with a 2 cm diameter, located in the proximal part of the rat's back. A perpendicular fasciocutaneous suture in the form of a loop was applied along the edges of the wound. The superficial fascia at the bottom of the wound was excised with perpendicular incisions, forming 5 x 5 mm cells. The resulting cells were sutured with U-shaped sutures.

The experimental study complied with the provisions of the European Convention for the Protection of Vertebrate Animals Used for Experimental and other Scientific Purposes (Strasbourg, 1986) and was approved by the Ethics and Bioethics Committee of Kharkiv National Medical University, Ukraine (Protocol N°17 dated 06-03-2024).

Photobiomodulation therapy was applied to the experimental group. The wounds were irradiated using the Lika-therapist M apparatus (Ukraine) with a wavelength of 660 nm, power of 10 mW, and energy density of 2 J/cm^2^, exposure time 628 s. The spot area was 3.14 cm^2^. The treatment was carried out continuously. The entire surface of the wound was irradiated the day after the wound was modeled and 48 hours later. The laser tip was held perpendicular to the wound surface at 3.53 cm to illuminate the entire wound surface.

The wound defects of the control group received a sham irradiation. The animals in the control group underwent the same manipulations as those in the experimental group, but with the device turned off. The duration of the manipulation was 628 s. The parameters of photobiomodulation therapy were selected based on literature data and our previous studies.

This therapy is effective in stimulating the healing process of skin wounds, with the best results achieved when using a wavelength of 658 nm ^16^. Energy density is also an important parameter. The flux density range of 0.5 to 2 J/cm^2^ demonstrates a peak effect on cell proliferation ^17^. The use of photobiomodulation therapy with a wavelength of 661 nm and an energy density of 2 J/cm^2^ accelerated wound healing and helped reduce inflammation in hairless mice ^18^.

Our previous study on the effect of photobiomodulation (wavelength 660 nm, energy density 0.5 J/cm^2^, power 10 mW) on wound defects showed positive results only in the early stages of wound healing ^19^. In this study, we decided to increase energy density. Our decision was supported by the positive results obtained when using a red laser with an energy density of 2 J/ cm^2^ for the treatment of bedsores ^20^.

Animals were sacrificed three days after chronic wounds were induced. Blood samples were collected by cardiac puncture.

According to the manufacturer's instructions, the levels of IL-1 ß, IL-4, IL-6, IL-10, TNF-α, bFGF, TGF-ß1, and ROS in blood serum were studied using ELISA. IL-1 ß, IL-4, IL-6, IL-10, TNF- α, and TGF-ß1 levels were determined using vector-best reagent kits (Ukraine). Reactive oxygen species and basic fibroblast growth factor levels were determined using eBioscience kits (USA).

The study of induced platelet aggregation was performed in platelet-rich plasma ^13^. Adenosine diphosphate (ADP) was used as an aggregation inducer at concentrations of 2.5 μmol/L, 5 μmol/L, and 10 μmol/L. The aggregation diagrams were recorded at 37 °C for 10 minutes. The degree of aggregation (maximum % light transmission of the plasma), time to reach the maximum aggregation rate (time to achieve the maximum % light transmission), and aggregation rate (calculated 30 seconds after the onset of platelet aggregation) were determined. The type of aggregation diagrams was also assessed.

A section of the animal's wound, including the central, main, and marginal sections, was excised for histological examination. The obtained material was embedded in paraffin and stained with hematoxylin and eosin and van Gieson's picrofuchsin. The preparations were analyzed and photographed using a PrimoStar microscope (Zeiss, Germany) and a digital camera Microocular. The degree of re-epithelialization, the number of polymorphonuclear leukocytes, fibroblasts, newly formed vessels, and collagen were assessed using a semiquantitative method, on a scale of 0, 1, 2, 3, 4 ^21^.

Statistical analyses were performed using the Statistica 12™ software package (StatSoft, USA). The effect of photobiomodulation on the parameters studied was evaluated using between-group statistical comparisons, as described below.

For each variable, normality of distribution was assessed using the Shapiro-Wilk test, as well as by visual inspection of Q-Q plots and boxplots. Homogeneity of variances was evaluated using Levene's test. When the assumptions for parametric analysis were met, between-group differences were assessed using one-way analysis of variance (ANOVA), followed by Tukey's post-hoc test. When these assumptions were not met, the nonparametric Kruskal-Wallis test was applied, followed by Dunn's post-hoc test with correction for multiple comparisons.

Given the small sample size, the results of parametric analyses were interpreted with caution. Data are presented as mean ± standard error of the mean (M ± Sß) or median and interquartile range (Me; Q_25_ to Q_75_) if non-normally distributed. A *p* < 0.05 was considered statistically significant. Histograms were plotted by GraphPad Prism 9™ (GraphPad Software, USA).

## Results

The concentrations of bioactive molecules in the animals' blood serum on day 3 of the experiment are presented in figure 1.

**Figure 1:**
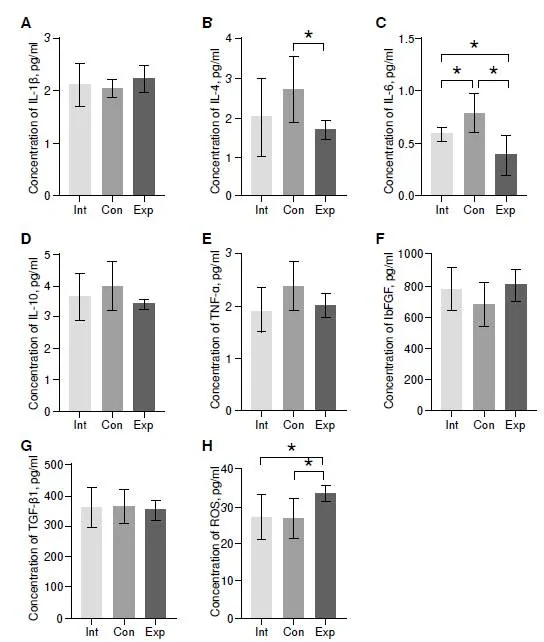
Changing the levels of the studied indicators in the animals' blood serum: (A) IL-1β, (B) IL-4, (C) IL-6, (D) IL-10, (E) TNF-α, (F) bFGF, (G) TGF-β1, (H) ROS; differences are significant relative to intact group and control group (*), (p < 0.05). The error bars represent the standard error of the arithmetic mean for each indicator (n = 6).

Int: intact group: Con: control group; Exp: experimental group

Our study showed that the concentration of IL-4 in the blood serum of animals undergoing photobiomodulation therapy decreased by 1.58 times in the experimental group compared to the control group (1.704 ± 0.095 versus 2.700 ± 0.355, respectively; p = 0.041). Additionally, on day 3 of the experiment, there were no significant differences in the levels of this cytokine between the intact and control groups, and between the intact and experimental groups.

In animals whose wound defects were treated with photobiomodulation therapy, a twofold decrease in serum IL-6 levels was observed on day 3 after surgery compared to similar indicators in the control group (0.395 ± 0.074 versus 0.789 ± 0.072, respectively; p = 0.0004).

The concentration of IL-6 in the control group increased by 1.33 times compared to intact group (0.789 ± 0.072 versus 0.592 ± 0.025, respectively; p = 0.038); in the experimental group, it decreased by 1.50 times compared to intact group (0.395 ± 0.074 versus 0.592 ± 0.025, respectively; p = 0.038).

Our study demonstrated that under the influence of photobiomodulation therapy on the third day of the experiment, the level of ROS in the blood serum of animals in the experimental group increased by 1.24 times compared to the control group (33.706 ± 0.897 versus 27.200 ± 2.072, respectively; p = 0.032) and 1.23 times compared to intact group (33.706 ± 0.897 versus 27.329 ± 2.489, respectively; p = 0.035). There was no difference between ROS levels in the control and intact groups.

On day 3 of the experiment, comparison of IL-1 ß, IL-10, TNF-α, bFGF, and TGF-ß1 levels between groups revealed no statistically significant differences. Indicators of the functional activity of platelets in animals at various concentrations of ADP are shown in tables 1-3.

**Table 1:** Indicators of the functional activity of platelets in animals on the 3 days of the experiment when using ADP at 2.5 μmol/L as an aggregation inducer; differences are significant relative to intact group (*) and control group (**), (p < 0.05); (n = 6).

Animals’ groups	Aggregation degree (%)	Aggregation time (s)	Aggregation rate (% / min at 30 s)
Intact	27.26 ± 2.44	61.17 ± 3.46	56.77 ± 2.05
Control	57.73 ± 3.06*	102.33 ± 7.22*	88.00 ± 2.79*
Experimental	13.75 ± 1.14*,**	59.50 ± 1.95**	46.30 ± 2.33*,**

**Table 2:** Indicators of the functional activity of platelets in animals on the 3 days of the experiment when using ADP at 5 μmol/L as an aggregation inducer; differences are significant relative to intact group (*) and control group (**), (p < 0.05); (n = 6).

Animals’ groups	Aggregation degree (%)	Aggregation time (s)	Aggregation rate (% / min at 30 s)
Intact	42.28 ± 4.54	108.17 ± 7.56	62.77 ± 4.87
Control	72.50 ± 0.44*	146.00 ± 5.25*	100.07 ± 2.72*
Experimental	36.45 ± 2.04**	91.50 ± 1.57*,**	59.90 ± 2.79**

**Table 3:** Indicators of the functional activity of platelets in animals on the 3 days of the experiment when using ADP at 10 μmol/L as an aggregation inducer; differences are significant relative to intact group (*) and control group (**), (p < 0.05); (n = 6).

Animals’ groups	Aggregation degree (%)	Aggregation time (s)	Aggregation rate (% / min at 30 s)
Intact	60.73 ± 1.45	150.83 ± 5.25	83.05 ± 4.46
Control	78.60 ± 1.18*	221.67 ± 7.86*	104.87 ± 3.01*
Experimental	53.00 ± 2.08*,**	155.00 ± 5.73**	71.70 ± 2.23*,**

A study of induced platelet aggregation showed that under the influence of photobiomodulation therapy on day 3 of the experiment, using ADP in concentrations of 2.5 μmol/L, 5 μmol/L, and 10 μmol/L as an aggregation inducer, all three indicators (aggregation degree, aggregation time, and aggregation rate) in the experimental group decreased compared to those in the control group.

In the control group, at all ADP concentrations, these three indicators were significantly higher than in intact animals. Macroscopically, after 3 days, the wounds in all animals were covered with a dense scab. Microscopic examination of wound site preparations in both groups revealed thickening of epidermal edges due to the proliferation of basal cells. This indicated the beginning of the process of wound defect reepithelialization.

In the control group, the wound cavity was filled with fibrin threads and detritus. The cellular component consisted mainly of neutrophilic granulocytes and macrophages. A significant number of erythrocytes at different stages of necrosis were noted because of bleeding after trauma. Reflecting the initial stage of the proliferative phase of wound healing, few fibroblasts were present (figure 2A).

**Figure 2:**
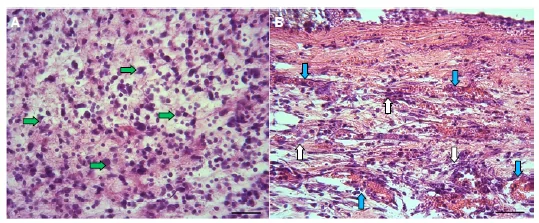
Wound site after 3 days in a rat: **A)** Control group: elements of inflammation and single fibroblasts (green arrows) on the background of fibrin threads. Hematoxylin and eosin, 40X. Scale bar: 50 μm. **B)** Experimental group: young granulation tissue with dilated full-blood capillaries (blue arrows), accumulations of fibroblasts and newly formed collagen fibers (white arrows). Hematoxylin and eosin, 40X. Scale bar: 50 μm.

In the experimental group, a layer of compacted fibrin with destroyed hematogenous elements was in the upper layer of the wound immediately below the scab. The wound defects were filled with predominantly young granulation tissue. The composition of the cellular component and extracellular matrix reflected intensely proliferative processes. Many fibroblasts, a few macrophages, and polymorphonuclear leukocytes were noted here. Collagen fibers were thin and chaotically arranged. Many newly formed full-blooded capillaries of vertical and horizontal courses were observed. Perivascular hemorrhages were a frequent phenomenon at most sites (figure 2B).

Semi-quantitative histological analysis showed that in the experimental group, the number of polymorphonuclear leukocytes decreased by 1.37 times, and the number of fibroblasts increased by 2.00 times, compared with the control group (p < 0.05) (figure 3). In addition, the experimental group showed an increase in the number of blood vessels and collagen compared to the control group, but the differences were not statistically significant.

**Figure 3:**
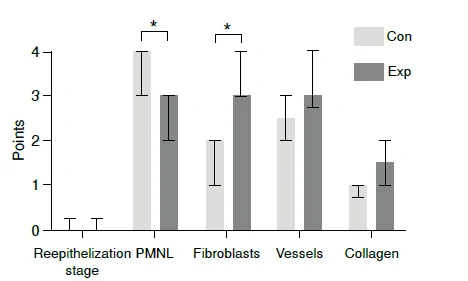
Results of semi-quantitative histologic analysis of wounds in rats after 3 days; differences are significant relative to the control group (*), (p < 0.05); bars and error bars represent median values and interquartile range for each indicator (n = 6).

Con: control group, Exp: experimental group

## Discussion

Tissue injury is known to stimulate the production of cytokines and chemokines by cells associated with the inflammatory response ^22^. Pro-inflammatory cytokines such as IL-1 ß, IL-6, and TNF-α promote cell recruitment to clear detritus and attract growth factors ^23^.

We did not observe any changes in the IL-1 ß and TNF- α levels at the parameters of the photobiomodulation therapy we selected. The use of this therapy in our study resulted in a decrease in the pro-inflammatory IL-6 level. This can be explained by the anti-inflammatory effect of laser radiation ^24^. It is known that in the early inflammatory phase, IL-6 modulates immune cell activation and local inflammation ^25^. Also, the decrease in IL-6 expression under the influence of photobiomodulation therapy may contribute to wound healing without any stimulus causing fibrosis ^26^. Similar results were obtained in our previous study ^27^.

According to the literature, the use of photobiomodulation with different energy densities led to different effects. Thus, laser therapy with an intensity of 0.84 to 1.4 J/cm^2^ increased the secretion of IL-6 and IL-8, whereas 1.97 J/cm^2^ suppressed the release of these cytokines in cells stimulated by lipopolysaccharide ^28^.

The histologic analysis performed in our work showed a lower number of macrophages and polymorphonuclear leukocytes in wound samples of the experimental group compared to similar studies in the control group.

Unlike pro-inflammatory cytokines, which help activate and recruit immune cells, anti-inflammatory cytokines help suppress excessive inflammation and promote tissue repair. Thus, IL-10 protects against tissue damage, balancing the inflammatory response ^29^. IL-4 is an anti-inflammatory cytokine that modulates macrophage polarization, enhances cellular proliferation, and supports tissue regeneration ^30^.

In our study, IL-10 levels remained unchanged under photobiomodulation therapy, possibly reflecting feedback regulation mechanisms. Similarly, exposure to laser irradiation at the selected parameters did not alter the levels of the potent anti-inflammatory cytokine IL-1 ß during the inflammatory phase. At the same time, a decrease in IL-4 levels was observed, which probably reflects the immunomodulatory effect of this therapy.

Photobiomodulation promotes balanced regulation of the inflammatory environment by reducing both excessive pro-inflammatory activity and anti-inflammatory signals to restore control over local inflammation. Given the nonlinear dose-response relationship, the selected therapy parameters may have caused moderate suppression of this cytokine.

TGF-ß1 functionality is also critical in the wound healing process, because its early release in the healing process stimulates the recruitment of inflammatory cells to the injury site. Our study showed no differences in TGF-ß1 levels in the experimental and control groups. Similar results were demonstrated in a study of the effect of photobiomodulation on wound healing in patients with aphthous stomatitis ^31^. It is also known that TGF-ß is a potent fibrogenic signal for fibroblasts, connective tissue, and epithelial cells, promoting the formation and remodeling of the extracellular matrix ^32^.

Data from our histological analysis demonstrates the presence of collagen fibers in the experimental group. We also observed a higher number of fibroblasts after exposure to photobiomodulation therapy.

This work studied basic fibroblast growth factor, which is involved in many biological reactions, including collagen deposition and angiogenesis, during the wound healing process. Exposure to photobiomodulation has shown trends towards an increase in this growth factor. The histological examination conducted in the study showed a doubling of the number of fibroblasts under the influence of photobiomodulation compared to the same indicator in the control group.

This basic fibroblast growth factor level can apparently be explained by the absence of the necessary further stimulation of fibroblasts. According to the literature, exposure of photobiomodulation therapy to an ischemic and delayed healing wound model in rats resulted in basic fibroblast growth factor gene expression promoting an increase in fibroblasts during the inflammation phase of wound healing ^33^.

In our study, a chronic wound model with hypoxia and impaired microcirculation was chosen. It is known that low and moderate physiological levels of ROS are beneficial for many pathophysiological processes involved in wound healing ^34^. A moderate increase in ROS levels is beneficial for destroying bacteria at the wound site and for revascularization. However, in large quantities, ROS leads to cell apoptosis ^35^.

A study of the modulation of ROS activity by photobiomodulation therapy demonstrated an increase in ROS levels in our work. Similar results were obtained in a study where photobiomodulation application increased ROS production in the cornea and vitreous. The increased ROS levels did not reach dangerous levels, confirmed by measuring lipid peroxidation levels ^36^.

Overall, a moderate elevation of ROS plays a crucial role in promoting platelet activation. Increased mitochondrial ROS contributes to a comparable decrease in platelet adhesion capacity and aggregation number ^37^. Our work has demonstrated a decrease in platelet aggregation activity at the inflammation stage, characterized by a decrease in the degree, time to reach the maximum rate of aggregation, and aggregation rate when using ADP at concentrations of 2.5 μmol/L, 5 μmol/L, and 10 μmol/L as an inducer of aggregation. This can be explained by the anti-inflammatory properties of laser therapy. The literature shows less intense aggregation in the animal group whose blood was exposed to photobiomodulation therapy during extracorporeal circulation compared to animals in the control group ^38^.

Preclinical studies are important in terms of safety screening and studying wound healing mechanisms ^39^. An animal model was chosen to study the effect of photobiomodulation on wound healing. Rats were selected for the study due to their cost-effectiveness and the possibility of obtaining samples from them, as well as the presumed similarity in wound healing rates among different animal species.

The models of chronic wounds in rats presented in this study have some limitations due to differences in physiology, anatomy, and repair mechanisms between rats and humans. The blood clotting time in rats is three times shorter, which contributes to rapid wound stabilization. Rapid reduction of the wound area is achieved by pulling the edges together, whereas in humans, wound closure occurs only through the process of re-epithelialization. Rats are also known to be less susceptible to infection and to be able to synthesize their own vitamin C, an essential cofactor for collagen production ^40^. Nevertheless, the experimental data obtained are of potential interest for subsequent clinical studies.

Currently, to improve the quality of extrapolation, correct interpretation, and comparability of data obtained in experimental models, standardized recommendations for presenting research results have been developed in the Wound Reporting in Animal and Human Preclinical Studies (WRAHPS).

One of the limitations of the present study is the small sample size (n = 6 per group), chosen in accordance with ethical principles aimed at minimizing the use of laboratory animals while ensuring scientific validity and appropriate statistical analysis. Another limitation is the short observation period (3 days), corresponding primarily to the inflammatory phase of the wound-healing process, which does not allow assessment of subsequent healing stages.

In addition, histological evaluation was performed without the use of specific cellular and molecular markers, and parallel measurement of serum and tissue biomarkers was not conducted, limiting the interpretation of cellular mechanisms and integration of local and systemic data. Finally, the study did not include comparisons with data from acute wounds nor assess the effects of variations in photobiomodulation parameters on reparative processes in chronic wounds, which restricts the ability to comprehensively evaluate therapeutic efficacy.

Photobiomodulation therapy at the inflammatory stage contributed to a moderate increase in the levels of active oxygen species while simultaneously reducing platelet aggregation activity and concentrations of proinflammatory cytokines, including IL-6 and IL-4. Histologically, a positive effect of this therapy on chronic wound healing was noted: reduced inflammation, accelerated transition to the proliferative phase, and active formation of fibroblasts. These data demonstrate the ability of photobiomodulation to modulate early inflammatory processes and stimulate regenerative mechanisms, which highlights its potential in the treatment of chronic wounds.

This study serves as a starting point for future experiments in this field. Further research plans include studying the expression of genes associated with inflammatory responses. Since the response to light exposure is nonlinear, additional studies are needed to investigate the effect of photobiomodulation therapy with different parameters on the regulation of chronic wound repair processes. Understanding the mechanisms of action of photobiomodulation and optimizing therapy parameters will create a basis for further improvement of treatment methods and their implementation in clinical practice.
